# Computational Study of the Influence of α/β-Phase Ratio and Porosity on the Elastic Modulus of Ti-Based Alloy Foams

**DOI:** 10.3390/ma16114064

**Published:** 2023-05-30

**Authors:** Claudio Aguilar, Javier Henriquez, Christopher Salvo, Ismelí Alfonso, Nicolas Araya, Lisa Muñoz

**Affiliations:** 1 Departamento de Ingeniería Metalúrgica y Materiales, Universidad Técnica Federico Santa María, Av. España 1680, Valparaíso 2390123, Chile; 2 Departamento de Ingeniería Mecánica, Facultad de Ingeniería, Universidad del Bío-Bío, Concepción 4081112, Chile; 3 Instituto de Investigaciones en Materiales, Unidad Morelia, Universidad Nacional Autónoma de México, Campus Morelia UNAM, Antigua Carretera a Pátzcuaro No. 8701, Morelia 58190, Michoacán, Mexico; 4 Departamento de Ingeniería de Materiales, Facultad de Ingeniería, Universidad de Concepción, Edmundo Larenas 315 Barrio Universitario, Concepción 4070409, Chile; 5 Instituto de Química, Facultad de Ciencias, Pontificia Universidad Católica de Valparaíso, Valparaíso 2373223, Chile

**Keywords:** Ti-6Al-4V foams, elastic modulus, modeling and simulation, microstructure

## Abstract

This work aims to perform a computational analysis on the influence that microstructure and porosity have on the elastic modulus of Ti-6Al-4V foams used in biomedical applications with different α/β-phase ratios. The work is divided into two analyses, first the influence that the α/β-phase ratio has and second the effects that porosity and α/β-phase ratio have on the elastic modulus. Two microstructures were analyzed: equiaxial α-phase grains + intergranular β-phase (microstructure A) and equiaxial β-phase grains + intergranular α-phase (microstructure B). The α/β-phase ratio was variated from 10 to 90% and the porosity from 29 to 56%. The simulations of the elastic modulus were carried out using finite element analysis (FEA) using ANSYS software v19.3. The results were compared with experimental data reported by our group and those found in the literature. The β-phase amount and porosity have a synergic effect on the elastic modulus, for example, when the foam has a porosity of 29 with 0% β-phase, and it has an elastic modulus of ≈55 GPa, but when the β-phase amount increases to 91%, the elastic modulus decreases as low as 38 GPa. The foams with 54% porosity have values smaller than 30 GPa for all the β-phase amounts.

## 1. Introduction

Titanium and its alloys are widely used in scientific and technological applications amongst various areas such as aerospace, nuclear, car, marine, and biomedical applications due to their high strength/density ratio, excellent corrosion resistance [1,2,3,4,5,6,7,8], and biocompatibility [9]. In the nuclear industry, they are used as a beam window material [10]. In this context, the most used alloy is Ti-6Al-4V alloy. For dental uses, this alloy undergoes a surface treatment to optimize contact between bone cells [11], as well as being used as orthopedic replacements due to its unique mechanical property combination (strength and ductility) [12].

Ti and Ti-based alloys exhibit an allotropy change at the β-transus temperature. Below the β-transus temperature, pure Ti and the great majority of its alloys exhibit a hexagonal close-packed (hcp) crystalline structure (called α-phase). Above this temperature, they exhibit a body-centered cubic (bcc) crystalline structure (called β-phase). The β-transus temperature for pure Ti is (882 ± 2) °C, which changes when alloying elements are added to Ti [13]. Many properties such as mechanical, chemical, and physical, along with others, are related to this allotropic phase transformation, which is very important for engineering applications. Ti-based alloys can exhibit various phases, therefore, being classified as (i) α-phase, (ii) α+β-phase, and (iii) β-phase, with further subdivision into near-α and metastable β alloys. The main characteristics of each group are (i) the α-phase alloys exhibiting a high elastic modulus, good creep resistance, good weldability, and excellent corrosion resistance, whereas (ii) the α + β-phase alloys possess high strength, good corrosion resistance, and moderate fracture toughness and weldability, their properties depending on the α/β relative proportion, and (iii) the β-phase alloys demonstrate high strength and fatigue resistance, lower elastic modulus, good formability, high hardenability, and moderate corrosion resistance [13,14,15,16]. Likewise, Ti-based alloys with metastable face-centered cubic (fcc or γ-phase) structures have been reported [17,18,19]. Two specific conditions are required when forming γ-phase, high deformation, and nanocrystalline grain size. The α- and β-phases are equilibrium phases while the γ-phase is a metastable phase [18,20,21,22,23,24,25,26].

The α-to-β-phase transformation can be promoted by controlling temperature, pressure, type, and amount of alloying elements [13]. At higher temperatures, the formation of the β-phase is promoted, and the pressure has a negligible effect on the transformation in pure Ti. Smith et al. [27] studied the effect that pressure has on Ti-Mo, Ti-24Nb-4Zr-8Sn, and Ti-36Nb-2Ta-0.3O alloys and observed that the β-phase is stable up to 40 GPa. When observing the p-T diagram of pure titanium [28], the β-phase is stable at high temperatures, and the α-phase transforms into the ω-phase at temperatures lower than 1155 K when the pressure increases. Alloying elements influence the β-transus temperature in different ways: α-stabilizers increase the β-transus temperature (typical elements in this group are Al, O, N, C [13]), whereas β-stabilizers move the β-transus towards lower temperatures (in this group are Ta, Nb, Z, W, V, Mo, Fe, Mn, Cr, Co, Ni, Cu, Si, H [13,29]). Certain alloying elements, such as Sn and Zr, show no influence on the β-transus [13]. Ti-based alloys can have two extreme microstructures, lamellar and equiaxial, both microstructures having a fine, as well as coarse, arrangement of α- and β-phases. The microstructure can be controlled by using thermo-mechanical methods. Ti-based alloys processed above the β-transus produce acicular or lamellar microstructures known as the β treatment structure [30]. The α-phase nucleates at grain boundaries of β-phase and then grows as lamellae. The thickness of lamellae depends on the cooling rate, where a slow rate produces coarse lamellar microstructures, and with a faster cooling rate, the β-phase may transform to martensite or a laths along with the retained β-phase [31]. If Ti-based alloys are treated below the β-transus, they exhibit a microstructure composed of both α- and β-phases. The equiaxial microstructures are produced by the recrystallization process in highly deformed samples [32].

Ti-6Al-4V alloy is widely used in biomedical applications since it has good mechanical/corrosion properties [8]. This alloy exhibits a microstructure α + β-phase [33], as well as having high specific strength (good mechanical strength-to-weight ratio), good corrosion resistance, and moderate fracture toughness and weldability, with their properties depending on the α/β relative proportion [8,15]. However, considering orthopedic applications, this alloy has a disadvantage, exhibiting a higher elastic modulus than bones, creating a mismatch between elastic modulus that produces the stress shielding effect [8]. The Young’s modulus of this alloy is around 112 GPa [8], and human bones are between 1 and 30 GPa [34].

Three routes can decrease the Young’s modulus of Ti-based alloys: (i) changing the nature of atomic bounds (solid solutions), (ii) introducing porosity into materials (synthesis of metallic foams), and (iii) synthesizing composite materials. The properties and performance of composite materials that could be used as orthopedic prostheses need more research, especially in load-bearing conditions [35]. The two first routes have been explored in biomaterials: (i) Using the first route, elastic modulus reduction through solid solution formation was not sufficient since the alloy exhibited values between 48 and 112 GPa [8]. This range of elastic modulus values is larger than that of a human bone, which ranges from 0.3 to 30 GPa [34,36,37,38]. (ii) Using the second route, elastic modulus values can be tailored to meet human bone requirements, and smaller values than 30 GPa can be obtained. For Ti-6Al-4V alloy foams, elastic modulus values of 7.5, 5.9, and 5.0 GPa for the porosity levels of 44.9, 50.1, and 56.2%, respectively, were measured by a compression test [39]. Guerra et al. [40] reported elastic modulus values of 5.6 and 3.2 GPa (measured using an ultrasound method) for 31 and 42% porosity, respectively, on Ti-6Al-4V foams that were synthetized using (NH_4_)_2_CO_3_ as a space-holder. Chen et al. [41] obtained a lower elastic modulus for Ti-6Al-4V foams via the electron beam melting method, in which the elastic modulus values were between 2.6 and 2.0 GPa for a porosity between 80.1 and 81.5%. Furthermore, Ti-6Al-4V foams synthetized by the selective laser melting method [41] exhibited a larger elastic modulus when compared to all Ti-based alloys. The latter is due to the pore shape being different from other foams, since they exhibited a diamond unit cell shape in the top and bottom views and an ellipsoid shape in the mantle view. Pore sizes were considered higher when compared to the rest of the ternary Ti-based alloys, being between 2500 and 4000 μm. The pore structure was designed using a Computer Aid Design (CAD) which allowed smooth surfaces to be obtained for the pore surfaces. The Gibson–Ashby model was applied, with the parameters being determined as α and n being equal to 1.5 and 2, respectively, in order to be acceptably fitted. On the other hand, Chen et al. [41,42] reported higher elastic values for foams having a lower porosity (43 to 71%), 55.9 to 9.7 GPa, respectively.

Based on the above, this work aims to analyze the influence that microstructure (α/β-phase ratio) and porosity have on the elastic modulus of Ti-6Al-4V alloy foam by modeling and simulation. The Ti-6Al-4V alloy was chosen because it has α + β microstructure and is an extensively used biomaterial. There are a significant number of works about experimentally measuring elastic modulus on pure Ti and Ti-based alloy foams, but there are fewer works related to simulations, with even fewer papers about simulations of the combined effect that microstructure (α/β-phase ratio) and porosity have on the elastic modulus of Ti-6Al-4V foams. This work is novel as it provides information on determining the elastic modulus as a function of the α/β-phase ratio and porosity. This information is expected to be useful in the design, synthesis, and heat treatment of titanium-based foams.

## 2. Computational Modeling and Simulation

### 2.1. RVE-FEM Method

For computational modeling and simulation, two methods were used: representative elementary volume (RVE) and finite element method (FEM). The models were designed in two steps: (a) generation of microstructure (α-phase matrix and β-phase matrix) and (b) addition of porosity to the models with microstructure.

(a) Microstructure Model Generation

**Figure 1:**
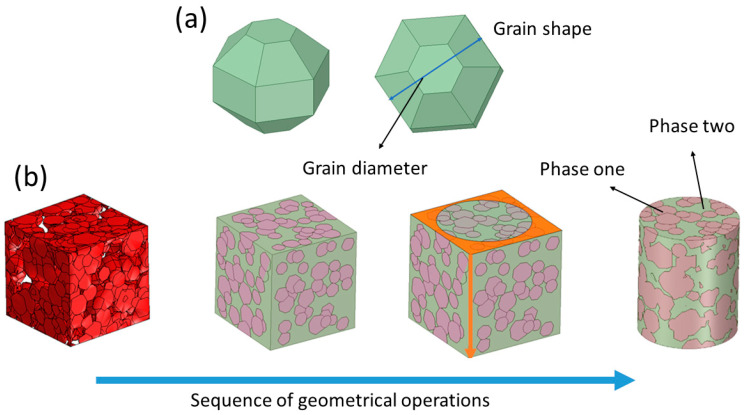
(**a**) Grain shape morphology and (**b**) step sequence used to obtain cylinder models with microstructures composed of α- and β-phases. Phase one can be α or β, and this is similar for phase two.

The microstructures were designed and generated by using following morphological parameters: (i) α-phase grains + intergranular β-phase (microstructure A), (ii) β-phase grains + intergranular α-phase (microstructure B), grain shape (truncated icosahedron, Figure 1a), orientation (random), grain size distribution (normal distribution), average mean grain size (50 μm), standard deviation (17 μm), model geometry (cylinder), height of the model (420 μm), and diameter of the model (350 μm). Microstructure parameters were taken from previous works of our group [40,43,44,45]. Both microstructures were obtained by varying the quantity of the α-phase and β-phase, as seen in Table 1. The sequence of steps used to obtain the final models is shown in Figure 1, the details of which are as follows:

(i) First step, using the parameters listed in Table 1, the microstructures were generated. The β-phase amount was variated from 0 to 90.5%. Differences between designed and obtained β-phase were observed, with the highest difference being seen in the designed β-phase, showing 40%. The RVE model was designed to be at least five times larger than grain sizes. The computational procedure consisted of periodically and randomly inserting volumes of grains into a matrix cube until the desired volumetric fraction was achieved. Grains are considered as one type of phase, and the space between grains (grain boundary) is considered as a second type of phase (Figure 1a).
(ii) Second step, the models were exported and loaded in the SpaceClaim CAD software (SpaceClaim Corporation, Version 2019 R3, Manufacturer, Ansys INC-SPACECLAIM CORP, Ciudad Concord, MA, USA).
(iii) Third step, using the draw options, the cubic model was transformed into the cylinder model, where colors represented each phase, α or β (Figure 1b).

(b) Porosity model generation

**Figure 2:**
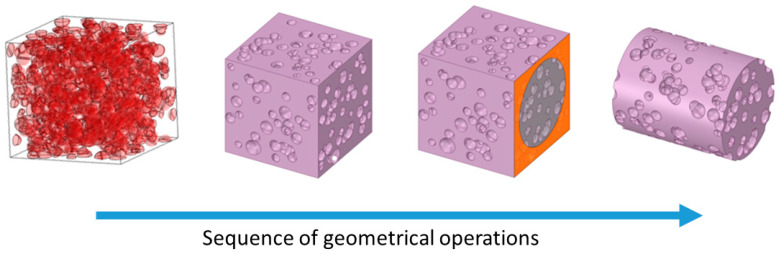
Sequence of steps to obtain cylinder models with porosity.

The morphological parameters used to design models with porosity are listed in Table 2. A homogeneous porosity distribution was used with a mean pore size of 250 μm. Cylinder models with dimensions of 2 mm in diameter and 2.4 mm in height were used. The porosity range was between 29 and 56 *v*/*v*%. A small difference between designed and obtained porosity values was observed, the highest difference being with the designed porosity of 56%. The sequence of steps to obtain models with porosity is shown in Figure 2. 

The details are as follows:

(i) First step, using the parameters listed in Table 2, pore models were generated. The RVE model was designed to be at least five times larger than pores [34,46,47].
(ii) Second step, the models were exported and loaded in the SpaceClaim CAD software.
(iii) Third step, using the draw options, the cubic model was transformed into cylinder models.
(iv) For each of the seven porosity models, the α/β ratio phase was added (Table 3). There are 7 porosity models and 11 microstructure models, which gives 77 configurations to be simulated. Porosity values were taken from porosity reported for human bones [48,49,50,51]. The simulation parameters used in this study are listed in Table 4. The elastic modulus values for the α-phase and β-phase were obtained from first-principles calculations made for Ti-xTM (TM = V, Cr, Nb, Mo) and ternary Ti-15TM-yAl alloys [52]. The calculated alloy compositions are similar to that of the Ti-6Al-4V alloy; thus, the elastic modulus values for the α-phase and β-phase were considered.

### 2.2. Image Analysis

The volumetric porosity was obtained by Equation (1) [55], where $V_{f}$ is the foam volume, and $V_{s}$ is the sample volume. All porosities were characterized by image analysis using ImageJ software. For each analysis, four images were used

(1) $$p=1-\rho_{app}=1-\frac{V_{f}}{V_{s}}$$

### 2.3. Simulations

The meshes were made using ten-node tetrahedron elements, with element sizes being chosen according to morphological scale and model size. The mesh refinement was verified with “Element Quality” and “Skewness” parameters. The boundary restrictions were (i) a compression uniaxial load applied in the cylinder upper face, (ii) a remote offset to dock the nodes of the top face in the same plane, displacement being free in the z-axis and zero in the x- and y-axes, with rotation being zero in all three axes, and (iii) a fixed support in the cylinder lower face. The applied load was fixed to obtain a stress of 10 MPa and applied to the cylinder upper face for all models. The elastic modulus values were determined using Equation (2), where $\sigma_{z}$ is the normal stress in the cylinder upper face, and $\varepsilon_{z}$ is the strain in the z direction (longitudinal). The $\varepsilon_{z}$ was determined by Equation (3), where $l_{f}$ and $l_{0}$ are the final and initial heights. The $\sigma_{z}$ was computed using Equation (4), where $F_{z}$ is the applied load in the z-axis, and $A_{z}$ is the cylinder upper face area.

(2) $$E_{z}=\frac{\sigma_{z}}{\varepsilon_{z}}$$

(3) $$\varepsilon_{z}=\frac{l_{f}-l_{0}}{l_{0}}$$

(4) $$\sigma_{z}=\frac{F_{z}}{A_{z}}$$

## 3. Results and Discussion

### 3.1. Effect of the Microstructure on E Values

**Figure 3:**
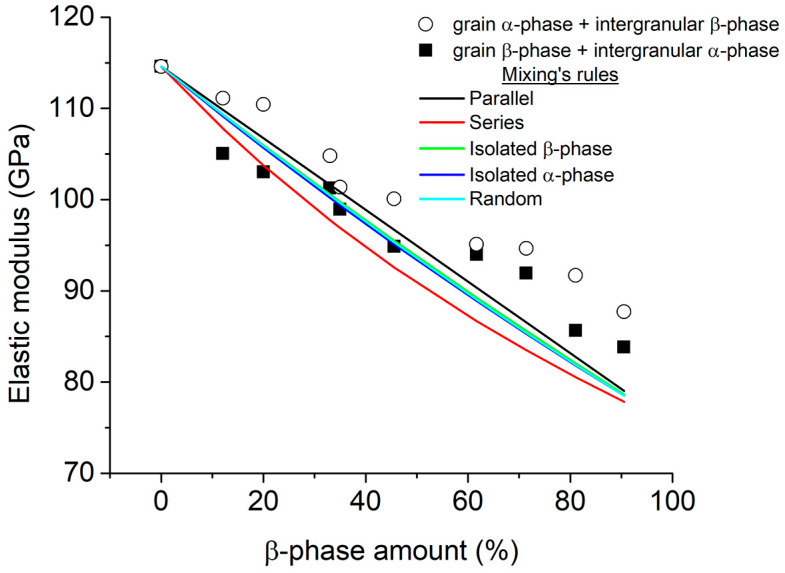
Variation of E as a function of β-phase amount for two conditions: (i) β-phase as matrix (microstructure B) and (ii) α-phase as matrix (microstructure A). Moreover, elastic modulus values were calculated using the mixing rules.

The influence that the α + β microstructure has on the elastic modulus of Ti-6Al-4V was simulated. The β-phase amount was changed from 0 to 90.5 *v*/*v*% (Table 1), and two microstructure types were simulated: (i) α-phase grains + intergranular β-phase (microstructure A) and (ii) β-phase grains + intergranular α-phase (microstructure B). For both microstructures, it was observed that the elastic modulus decreased as a function of the β-phase amount, from 115 GPa (0% β-phase) to ≈88 and 84 GPa for microstructures A and B, respectively (90.5% β-phase), which can be seen in Figure 3. It is observed that all elastic modulus values for microstructure B were smaller when compared to microstructure A for all β-phase amounts. The elastic modulus variation for both microstructures is compared with simple calculations on the basis of the material mixing rules that consisted of two phases (α- and β-phases): (a) parallel, Equation (5), (b) series, Equation (6), (c) isolated β-phase, Equation (7), (d) isolated α-phase, Equation (8), and (e) random, Equation (9) [56], where $E_{\alpha }$ and $E_{\beta }$ are the elastic modulus for both α- and β-phases, and $V_{\alpha }$ and $V_{\beta }$ are the volume fraction of the α- and β-phases, respectively. It was observed that the E values obtained by FEM and the mixing rules were no closer, showing that none of the mixing rule models used captured the microstructure phenomenology.

(5) $$E_{alloy}=E_{\alpha }x_{\alpha }+E_{\beta }x_{\beta }$$

(6) $$\frac{1}{E_{alloy}}=\frac{V_{\alpha }}{E_{\alpha }}+\frac{V_{\beta }}{E_{\beta }}$$

(7) $$E=\frac{V_{\alpha }E_{\alpha }+V_{\beta }E_{\beta }\left(\frac{3E_{\alpha }}{2E_{\alpha }+E_{\beta }}\right)}{V_{\alpha }+V_{\beta }\left(\frac{3E_{\alpha }}{2E_{\alpha }+E_{\beta }}\right)}$$

(8) $$E_{alloy}=\frac{V_{\beta }E_{\beta }+V_{\alpha }E_{\alpha }\left(\frac{3E_{\beta }}{2E_{\beta }+E_{\alpha }}\right)}{V_{\beta }+V_{\alpha }\left(\frac{3E_{\beta }}{2E_{\beta }+E_{\alpha }}\right)}$$

(9) $$V_{\alpha }\left(\frac{E_{\alpha }-E_{alloy}}{E_{\alpha }-2E_{alloy}}\right)+V_{\beta }\left(\frac{E_{\beta }-E_{alloy}}{E_{\beta }-2E_{alloy}}\right)=0$$

**Figure 4:**
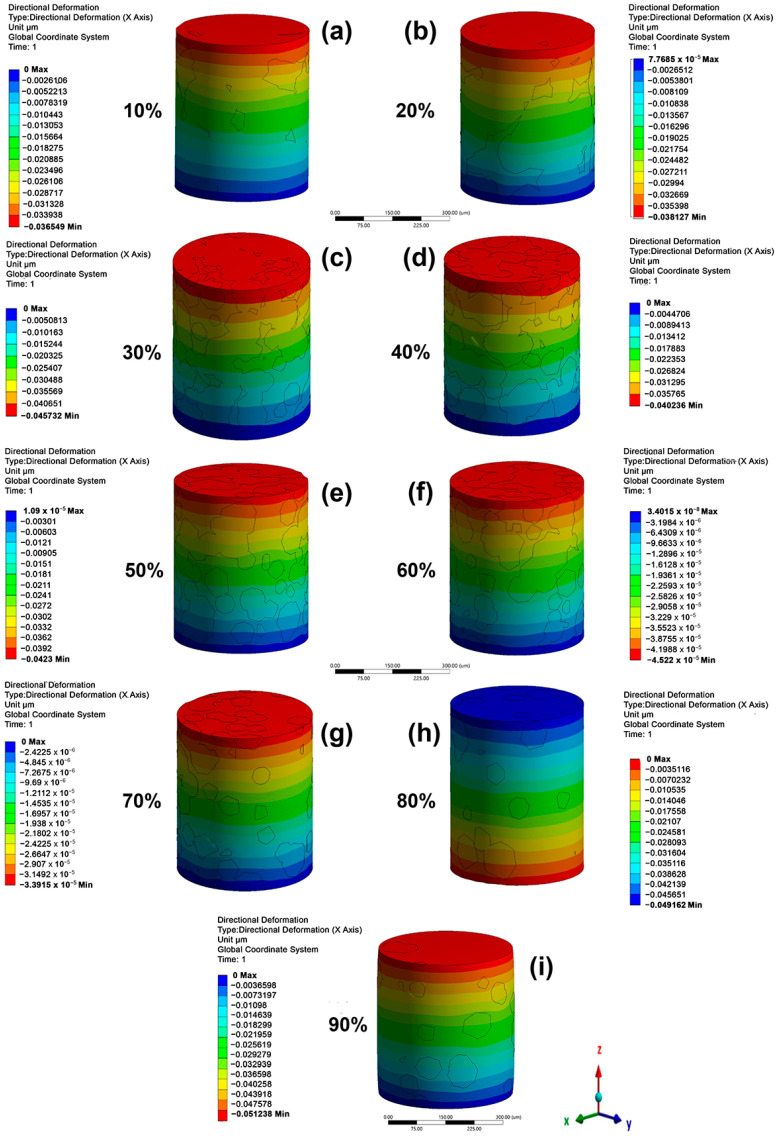
Directional deformation in height (in mm) under compression for the bulk samples with β-phase amounts of (**a**) 10%, (**b**) 20%, (**c**) 30%, (**d**) 40%, (**e**) 50%, (**f**) 60%, (**g**) 70%, (**h**) 80%, and (**i**) 90% for a microstructure composed as α-phase grains + intergranular β-phase (microstructure A).

**Figure 5:**
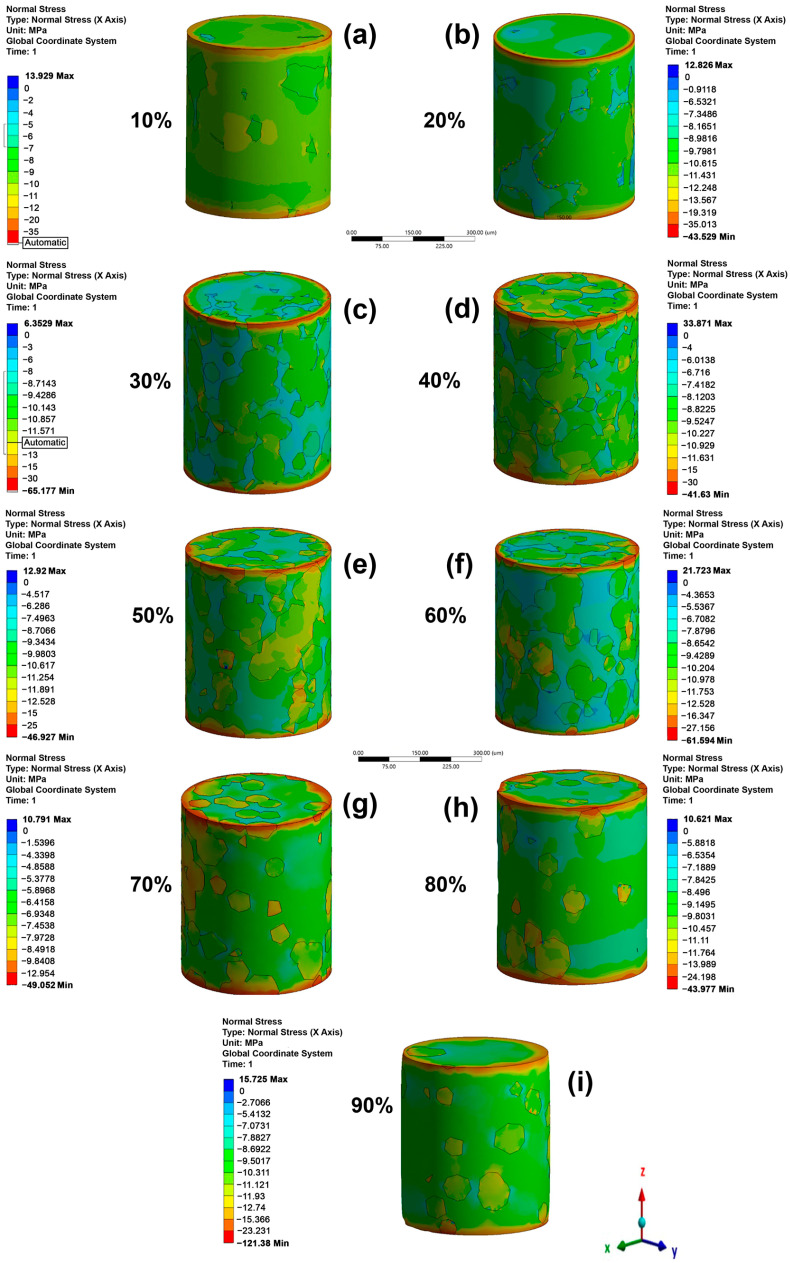
Directional stress in height (in mm) under compression for the bulk samples with β-phase amounts of (**a**) 10%, (**b**) 20%, (**c**) 30%, (**d**) 40%, (**e**) 50%, (**f**) 60%, (**g**) 70%, (**h**) 80%, and (**i**) 90% for a microstructure composed as α-phase grains + intergranular β-phase (microstructure A).

The directional displacement for the cylindrical models under the microstructure A loads (for all of β-phase amounts) is shown in Figure 4. The results for microstructure B are not shown in the section for simplicity. The analysis for both microstructures is similar because the models used were the same for microstructure A. The higher directional displacements are seen as negative since compression stresses were applied and were proportional to the α/β-phase ratio. The directional displacements showed higher increases when the α/β-phase ratio decreased, and this is because the β-phase has a smaller elastic modulus than the α-phase. Figure 5 shows the stress distribution for all samples as a function of the β-phase amount for microstructure A. A higher stress is observed in the α-phase than in the β-phase since the first phase has a higher elastic modulus. The edges of the top and bottom side show higher compressive stresses (red color) due to movement restriction, simulating the effect of the compression plates. It is noted that when the β-phase amount is less than 30%, its irregular morphology causes a reduction in the load transfer. The applied load is transferred by continuous grain α-phase. When the β-phase amount is higher than 30%, it begins to continuously promote an elastic modulus reduction.

The elastic modulus of Ti-based alloys depends on chemical composition, microstructure, and the synthesis method. For the Ti-6Al-4V alloys with an α-β microstructure, several elastic modulus values have been reported, such as wrought Ti-6Al-4V ELI and standard-grade Ti-6Al-4V ELI exhibiting values of 110 and 112 GPa, respectively [8]. Cho et al. [57] obtained Ti-6Al-4V alloys by a selective laser melting method and analyzed microstructural inhomogeneity and variations in elastic modulus. They observed that the microstructure was composed of an α-phase with smaller β-phase amounts containing α-β lamellae, where the β-phase forms at the α-phase boundaries as ultrafine strips. In some samples, the presence of α′-phase was found. They reported elastic modulus values too high to be used as biomedical material, between 120 and 180 GPa. Gain et al. [58] obtained Ti-6Al-4V alloys by casting, hot rolling, and annealing processes. They observed a microstructure of an α-phase grain with β-phase submicron size grain uniformly distributed in the α-phase matrix. An elastic modulus of 107.3 GPa was measured. Zhou et al. [59] obtained the Ti-6Al-4V alloy by a selective laser melting method and reported a microstructure composed of α-phase with minimal β-phases and α′-phase. The elastic modulus reported was 107 GPa.

### 3.2. Effect That Microstructure and Porosity Have on E Values

**Figure 6:**
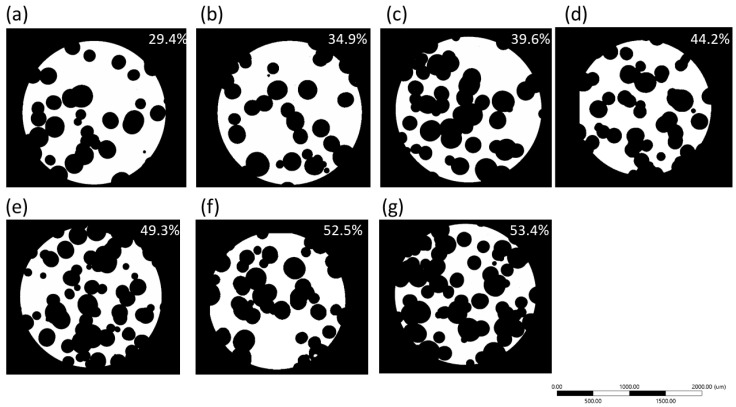
Transversal images of different models showing the pore coalescence. (**a**) 29.4%, (**b**) 34.9%, (**c**) 39.6%, (**d**) 44.2%, (**e**) 49.3%, (**f**) 52.5%, (**g**) 53.4%.

**Figure 7:**
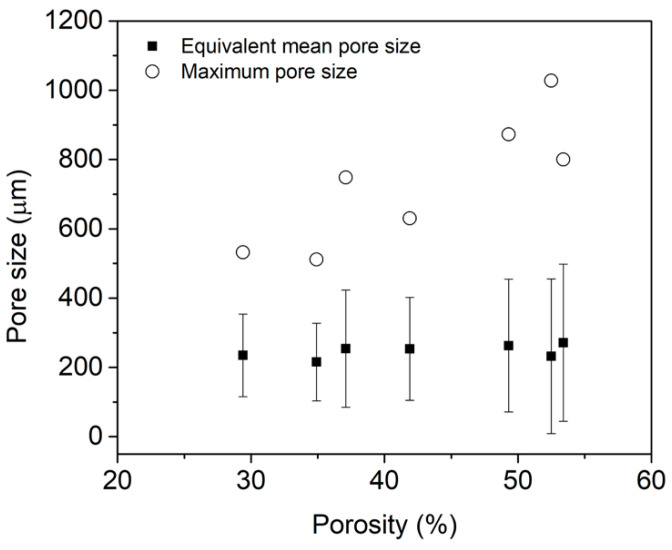
Equivalent mean pore size and maximum pore size variation with porosity.

**Figure 8:**
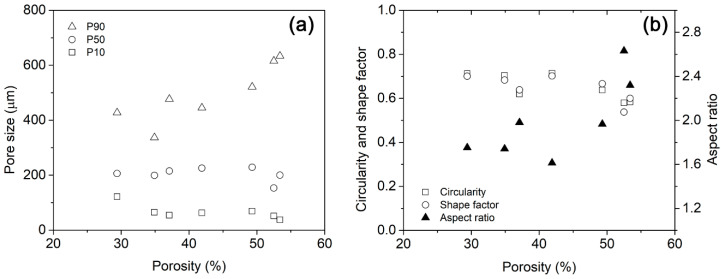
(**a**) Equivalent mean pore size and maximum pore size variation and (**b**) pore shape characterization, circularity, shape factor, and aspect ratio as a function of porosity.

Seven models with different porosity values were generated according to the procedure given by Figure 1 and Figure 2. When porosity values increased, they produced a pore coalescence, increasing the pore size and influencing the final porosity value of the foams (Figure 6). Using the transversal image of the foams, a morphological analysis was performed to characterize the pore coalescence. The equivalent mean pore size and maximum pore size for each model was determined (Figure 7). The mean equivalent pore size had a slight porosity change, but all of them were within the standard deviation, meaning that all were equal (around 246 μm). The standard deviation of the mean equivalent pore size and the maximum pore size increased for high porosity values due to the pore coalescence. The maximum pore size increased from 511 to 1027 μm. Small porosity value differences were observed between pores designed and obtained, and all differences were smaller than 2.5% (Table 3). Those porosity differences are acceptable to simulations. A pore size distribution can be easily described using three parameters, which were taken from pore-based cumulative pore size distribution at three sizes, P10, P50, and P90. Those parameters corresponded to the pore sizes at 10, 50, and 90% on the cumulative distribution. Figure 8a shows the variation of the equivalent mean pore size as a function of porosity level for the statistical parameters P10, P50, and P90. The P90 values increased with the porosity, showing that higher pore sizes existed at high porosity values. The P50 values were relatively constant with porosity, and the P10 values showed a diminution with the porosity, meaning that at high porosity the number of pores with small sizes was less. The pore shape was characterized using three dimensionless parameters: (i) the circularity (C), Equation (10), where A is the area of the pore, and P is the perimeter of the pore; (ii) the shape factor (F_f_), Equation (11); and (iii) aspect ratio (AR), Equation (12), where x_min_ and x_max_ are the smallest and largest pore size dimensions. The circularity and shape factor decreased when porosity increased from 0.712 (C), 0.700 (Ff) for a porosity of 29% to 0.583 (C), 0.600 (Ff) for a porosity of 53.4%, respectively (Figure 8b). The aspect ratio values changed from 1.752 to 2.319, and this is explained by the pore coalescence producing elongated pores (Figure 6).

(10) $$C=\left(\frac{4\pi A}{P^{2}}\right)$$

(11) $$F_{f}=\left(\frac{4\pi A}{P^{2}}\right)$$

(12) $$AR=\frac{X_{max}}{x_{min}}$$

**Figure 9:**
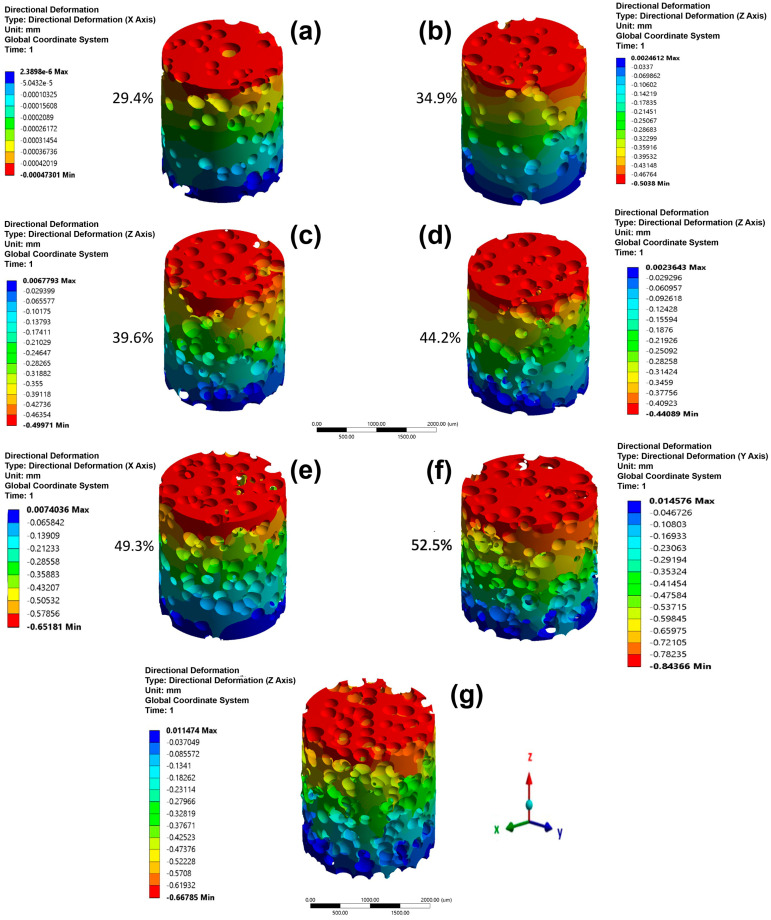
Directional deformation in height (in μm) under compression for the foam samples with 0% β-phase amount. (**a**) 29.4%, (**b**) 34.9%, (**c**) 39.6%, (**d**) 44.2%, (**e**) 49.3%, (**f**) 52.5%, (**g**) 53.4%.

**Figure 10:**
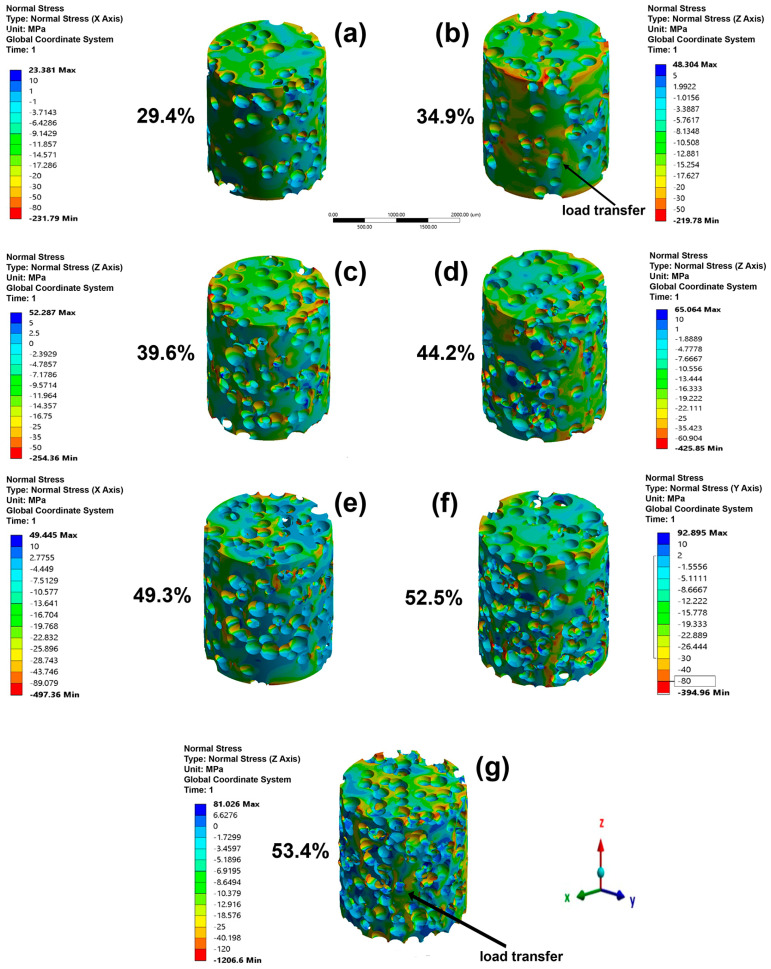
Directional stress in height (in mm) under compression for the foam samples with 0% β-phase amount. (**a**) 29.4%, (**b**) 34.9%, (**c**) 39.6%, (**d**) 44.2%, (**e**) 49.3%, (**f**) 52.5%, (**g**) 53.4%.

As was mentioned, seven foam models were made and used in simulations with different β-phase amounts. For space reasons, only the graphical responses of the foams with 0% β-phase are shown in this section. Directional displacements in height as a function of porosity are shown in Figure 9. The negative displacement values are due to a compressive load being applied. Directional displacement distributions were observed for all foams, which is due to the random presence of pores. Higher displacements were produced in regions closer to big pores produced by coalescence. The maximal displacement values increased as a function of the porosity. The top side of the cylinders exhibit higher displacement values for all foams because of loads being applied there. The stress distribution as a function of porosity is shown in Figure 10. The higher compressive stress values are observed close to two regions, the cylinder edge and the pore edge (red area). It can be seen that the pores act as stress concentrators, increasing this accumulation in the thinner and more vertical walls of the models. In the high porosity level model, the magnitude of the maximum stress increased considerably due to the increase in stress concentrators. However, the number of elements that exhibit stress concentration is less than 1%. This can be verified by comparing the stress value of each element, which shows that most of them have stress values around −1206 to 81 MPa for the foam with higher porosity level (53.4%). The number of elements with higher compressive stress are very low or negligible; therefore, they do not significantly affect the elastic modulus value. Moreover, regions with tensile stress were observed, which increased with the porosity. The maximum tensile stress values were smaller than the compressive stresses, 23 MPa (tensile stress)/−231 MPa (compression stress) and 81 MPa (tensile stress)/−1206 MPa (compression stress) for porosities of 29.4 and 53.4%, respectively. The absolute value of the maximum compression stress was 10 times higher than the absolute value of the maximum tensile. The tensile stresses were located in regions between pores. The load transfer was better in regions with or without less presence or pores, as shown by the arrows in the models with 34.9 and 53.4% porosity. The best load transfer is demonstrated using a green color scale (Figure 10b,g).

**Figure 11:**
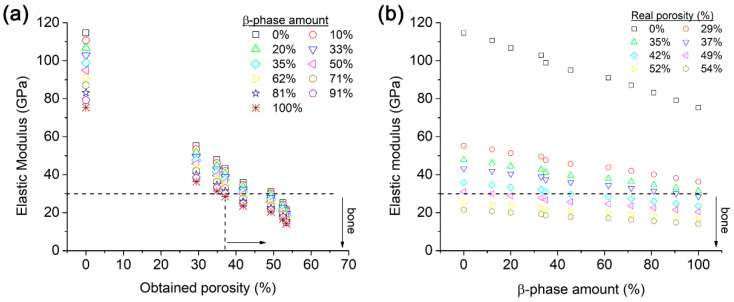
Variation of elastic modulus as a function of (**a**) porosity and (**b**) β-phase amount.

The majority of elastic modulus values obtained by simulation for Ti-based alloy foams reported in the literature are about homogeneous porosity. The elastic modulus simulated in this work for the Ti-6Al-4V foams decreased when porosity levels increased for all β-phase amounts (Figure 11a). It is noticeable that an elastic modulus smaller than 30 GPa that is required for biomedical uses was obtained for a high β-phase amount and porosity. The β-phase amount and porosity have a synergic effect on the elastic modulus; for example, when the foam has a porosity of 29% and 0% β-phase, it has an elastic modulus of ≈55 GPa, but when the β-phase amount increases to 91%, the elastic modulus reduces to as low as 38 GPa (Figure 11a). The results show an exponential decrease in the elastic modulus with porosity (Figure 11a) and with the β-phase (Figure 11b). The Ti-6Al-4V foams that exhibited an elastic modulus smaller than 30 GPa had a ≥37% porosity value (Figure 11a). For a porosity value of 37.1%, the foams with an elastic modulus smaller than 30 GPa have β-phase amounts of 91 and 100%, but all foams with 54% porosity as well as all β-phase amounts have values smaller than 30 GPa (Figure 11a). The same results are shown as a function of the β-phase amount (Figure 11b). There are two mechanisms that act to reduce the elastic modulus of foams. First, foams are composed of two crystal structures, α- and β-phases, with different elastic modulus values (Table 4). Therefore, the elastic modulus of the foams is influenced by the specific elastic modulus and amount of each phase. When the amount of the β-phase increases, the elastic modulus of the foam decreases because the β-phase has a smaller elastic modulus than the α-phase. Second, the elastic modulus is a measure of a material’s stiffness or resistance to deformation under an applied force. When a load is applied to a metal or alloy, the atoms within the material move closer together, causing a change in the material’s shape. However, in a metallic foam, the presence of pores within the material allows for more movement and deformation, as the load can be absorbed by the surrounding pores. As a result, the effective stiffness of the material decreases as the porosity increases, leading to a smaller elastic modulus. The porosity also decreases the density of the material, which can further reduce the elastic modulus.

**Figure 12:**
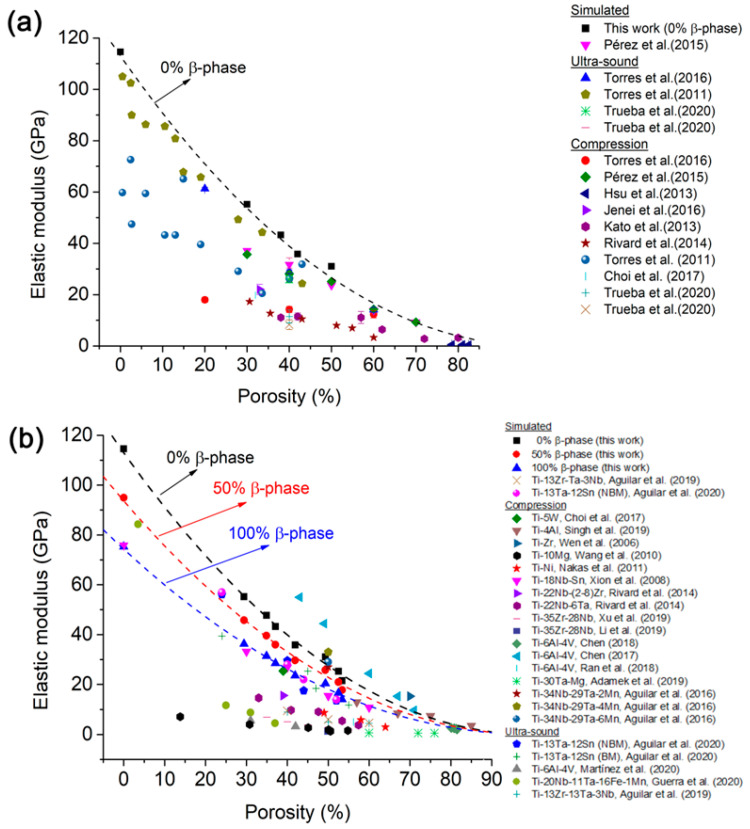
Comparison of elastic modulus simulated with elastic modulus values reported in the literature: (**a**) pure Ti foams and (**b**) Ti-based alloy foams [34,36,37,39,40,41,42,55,60,62,63,64,68,69,70,79,80,81,82,83,84,85,86,87,88].

There are many works about experimentally measuring the elastic modulus on pure Ti and Ti-based alloy foams, but there are few works related to simulations and even fewer papers about simulations of the combined effect that microstructure (α/β-phase ratio) and porosity have on the elastic modulus of Ti-6Al-4V foams. Figure 12 shows a comparison between several elastic moduli of pure Ti foams and Ti-based alloy foams, where it is possible to observe the following aspects: (i) elastic modulus decreases when the quantity of porosity increases, (ii) elastic modulus values measured by compression test are higher than values obtained by ultrasound method, and (iii) elastic modulus values show a high data dispersion as a function of porosity. This data dispersion is influenced by several variables that depend on the phase microstructure, morphology of pores, foam manufacturing method, and methods used to measure elastic modulus values. The major number of reports are based on homogeneous porosity [55,60,61,62], and there are very few works about graded porosity distribution [63,64].

Figure 12a shows the variation of elastic modulus with porosity for pure Ti foams reported in the literature and values obtained in this work by simulations. The elastic modulus variation simulated different pure Ti foams with 0% β-phase (or 100% of an α-phase, because pure Ti has only an α-phase) is included (full black square). The simulated E values are in the higher range values compared with the experimental E values reported. This is because the elastic modulus obtained by simulations uses models with a determined porosity value, and E values are influenced only by a tailored porosity. On the other hand, there are additional factors influencing the E values of foams experimentally synthesized, such as process inefficiency, type of processing, size and size pore distribution, pore shape, interconnectivity, shape factor, pore roughness, and wall thickness [65,66]. Torres et al. [60,61] measured elastic modulus by compression and ultrasound methods, reporting that the elastic modulus measured by the compression technique was smaller than those measured by the ultrasound method. Manonukul et al. [67] determined that the experimental modulus of elasticity oscillates between ~0.40 and ~0.62 GPa for cp-Ti foams with porosity levels between ~86.5 and ~91.2 %. Hsu et al. [55] measured the elastic modulus (~0.43 to ~0.53 GPa) for pure Ti foams with porosities between ≈83 and 79%. Rivard et al. [68] measured the elastic modulus of cp-Ti foams ranging between 17 and 3 GPa for porosity values of 30 to 60%. They reported a shape factor smaller than ~0.62, where pores were irregular and elongated, with the elastic modulus also being measured by a compression test. Pérez et al. [37] reported simulated values of ≈37 and ≈32 GPa for 30 and 40% porosity, respectively.

The variation of elastic modulus values reported for several Ti-based alloy foams as a function of porosity is shown in Figure 12b. Elastic modulus values simulated for the three Ti-6Al-4V foams had 0, 50, and 100 % β-phase added to them. In general terms, two important features can be observed: (i) the elastic modulus values simulated are higher than the experimental E values reported in the literature, and (ii) the experimental elastic modulus exhibits a high dispersion, which is strongly influenced by measurement method variables (type and calibration of equipment), processing variables of foams (temperature, pressure, atmosphere, type of process), and materials variables (type and amount of alloying elements, matrix phase type, pore size and shape, pore size distribution, microstructure, α/β-phase ratio). The simulated elastic modulus values are acceptable because they are within all experimental values reported by other works. Chen et al. [41] measured very small values of 2.6 to 2.0 GPa (compression test) for porosities around 80.1–81.5% for Ti-6Al-4V foams obtained by a selective laser melting method. Rivard et al. [68] studied the effect that the quantity of Zr has on the elastic modulus of Ti-22Nb-(2-8)Zr alloy foam. They measured this by compression test and reported values of 1.5 to 16 GPa for a variation of porosity from 67 to 15%, respectively. Xiong et al. [69] measured an elastic modulus ranging between 10.8 and 33.2 GPa for porosities of 60 and 30%, respectively, for Ti-18Nb-4Sn (wt.%) foams. Moreover, the work of Chen et al. [42] reported higher elastic values for foams with a lower porosity (43 to 71%), 55.9 to 9.7 GPa, respectively. Guerra et al. [40] reported elastic modulus values of 5.6 and 3.2 GPa (measured by ultrasound) for porosities of 31 and 42%, respectively, for Ti-6Al-4V foams synthesized using a space-holder method, and Aguilar et al. [70] measured values of 56.1 to 13.4 GPa (ultrasound method) for a porosity range of 24 to 52% for Ti-13Ta-12Sn alloy foams.

It should be mentioned that there are analytical models that can be used to determine the elastic modulus, such as Gibson and Ashby [71], the Spriggs model [72], Warren and Kraynik [73], the Nielsen model [74], the Pabst and Gregorova model [75], the Zhu model [76], the Knudsen model [77], and the Phani and Niyogi model [78]. All of these models use equations based on porosity or relative density. They can be used in two ways: (i) as a first approach to estimate the elastic modulus using preliminary assumptions or (ii) after the porosity parameters have been determined (by simulation or measurement) to save time in the elastic modulus calculation, as they are easy to use. An example of the Gibson and Ashby model is shown in Figure 12a,b (dotted line), which gives reasonable results of the variation of E versus porosity for different amounts of the β-phase. It is not the goal of this work to calculate the elastic modulus using all analytical models mentioned; therefore, no values are given in this regard. However, the equations of analytical models are given in Appendix A for the reader’s utility.

## 4. Conclusions

In this work, a computational study of the influence that microstructure and porosity have on the elastic modulus of Ti-6Al-4V foams was made. Based on this, it can be concluded:

(a) Microstructure.

For both microstructures, it was observed that the elastic modulus decreases as a function of the β-phase amount, from 115 GPa (0% β-phase) to ≈88 and 84 GPa (90.5% β-phase) for microstructures A and B, respectively. None of the mixing rule models used captured the microstructure phenomenology; therefore, there are differences between the values obtained by FEM.

It was observed in the α-phase (E = 114.6 GPa) that there were higher stresses than in the β-phase (E = 75.3) because the α-phase has a higher elastic modulus. The applied load is transferred by a continuous phase (α-phase or β-phase).

(b) Porosity.

The β-phase amount and porosity have a synergic effect on the elastic modulus; for example, when the foam has a porosity of 29% and 0% β-phase, it has an elastic modulus of ≈55 GPa, but when the β-phase amount increases to 91%, the elastic modulus is 38 GPa. Foams that have 54% porosity have values smaller than 30 GPa for all the β-phase amounts.

It can be seen that the pores act as stress concentrators, which increases stress accumulation in the thinner and more vertical walls of the models. However, the number of elements that exhibit stress concentration is less than 1%. Therefore, they do not significantly affect the elastic modulus value.

In summary, a reasonable estimation of the elastic modulus of foams can be obtained by using the information given in Figure 11, which considers the α/β-phase ratio and porosity. This information is useful for the design, synthesis, and heat treatments of titanium-based foams.

## Figures and Tables

**Table 1:** Quantity of the designed and obtained β-phase.

Designed β-Phase Amount (*v*/*v*%)	Obtained β-Phase Amount (*v*/*v*%)	Difference between Designed and Obtained β-Phase
10	12.0	2.03
20	20.0	0.
30	33.2	3.2
40	34.7	−5.3
50	45.5	−4.5
60	61.6	1.6
70	71.3	1.3
80	81.4	1.4
90	90.5	0.5

**Table 2:** Morphological parameters of pores.

Morphological Parameters	Characteristic of Porosity
Distribution of porosity	Homogenous
Shape pore	Sphere-cylinder
Aspect ratio	1.1
Distribution pore function	Normal distribution
Average pore size	250 μm
Standard deviation	50 μm

**Table 3:** Comparison between designed and obtained porosity values.

Designed Porosity (*v*/*v*%)	Obtained (Or Real) Porosity (*v*/*v*%)	Difference between Designed and Obtained Porosity
29	29.4	0.4
34	34.8	0.8
38	37.0	−1.0
43	41.8	−1.2
47	49.2	2.2
52	52.5	0.5
56	53.4	−2.6

**Table 4:** Mechanical parameters used in the simulations [52].

Phase	Density, g/cc	Elastic Modulus, GPa [52]	Poisson’s Ratio [37,53,54]
α	4.51	114.6	0.33
β	4.51	75.3	0.33

## Data Availability

The raw/processed data required to reproduce these findings cannot be shared at this time due to technical or time limitations.

## Appendix A

### Analytical Models to Determine E Values

Gibson and Ashby model [71].

This model is given by Equation (A1), where ρ and ρ_0_ was considered the foam and bulk material density, respectively, and α and n are parameters that depend on pore structure. Those parameters vary as α between 0.1 and 4 and *n* between 1.8 and 2.2.

(A1) $$E=\alpha E_{0}{\left(\frac{\rho }{\rho_{0}}\right)}^{n}$$

Knudsen–Spriggs model [72,77].

This model is given by Equation (A2), where p is the porosity, and b is parameter related to particles stacking.

This expression cannot be used when *p* = 1 since it does not satisfy the condition that the elastic modulus value must be included so that it is equal to zero.

(A2) $$E=E_{0}\;e^{-bp}$$

Phani–Niyogi model [78].

This model is given by Equation (A3), where p is the porosity, p_c_ is the critical porosity at which E = 0, and *m* is a constant that depends on pore distribution geometry.

(A3) $$E=E_{0}{\left(1-\frac{\rho }{\rho_{c}}\right)}^{m}$$

Pabst–Gregorová [75].

This model is given by Equation (A4), where p is the porosity, p_c_ is the critical porosity, and *a* is a constant that depends on the packing geometry factor (*a* is equal to 1 for spherical-shaped pores).

(A4) $$E=E_{0}\;\left(1-a\;p\right)\left(1-\frac{P}{P_{c}}\right)$$

Nielsen [74].

This model is given by Equation (A5), where F_f_ = 4pA/PE^2^, A is the pore area, and PE is the experimental perimeter of the pore.

(A5) $$E=E_{0}\;\left[\frac{{\left(1-p/100\right)}^{2}}{1+\left(\left(\frac{1}{F_{f}}-1\right)p/100\right)}\right]$$
